# Molecular Mechanisms and Emerging Therapeutic Targets of Triple-Negative Breast Cancer Metastasis

**DOI:** 10.3389/fonc.2018.00031

**Published:** 2018-02-22

**Authors:** Christiana Neophytou, Panagiotis Boutsikos, Panagiotis Papageorgis

**Affiliations:** ^1^Department of Biological Sciences, School of Pure and Applied Sciences, University of Cyprus, Nicosia, Cyprus; ^2^Department of Life Sciences, European University Cyprus, Nicosia, Cyprus

**Keywords:** triple-negative breast cancer, metastasis, targeted therapy, tumor microenvironment, dormancy

## Abstract

Breast cancer represents a highly heterogeneous disease comprised by several subtypes with distinct histological features, underlying molecular etiology and clinical behaviors. It is widely accepted that triple-negative breast cancer (TNBC) is one of the most aggressive subtypes, often associated with poor patient outcome due to the development of metastases in secondary organs, such as the lungs, brain, and bone. The molecular complexity of the metastatic process in combination with the lack of effective targeted therapies for TNBC metastasis have fostered significant research efforts during the past few years to identify molecular “drivers” of this lethal cascade. In this review, the most current and important findings on TNBC metastasis, as well as its closely associated basal-like subtype, including metastasis-promoting or suppressor genes and aberrantly regulated signaling pathways at specific stages of the metastatic cascade are being discussed. Finally, the most promising therapeutic approaches and novel strategies emerging from these molecular targets that could potentially be clinically applied in the near future are being highlighted.

## Introduction: Tumor Heterogeneity and Current Challenges in Triple-Negative Breast Cancer (TNBC) Treatment

Breast cancer is the most frequently diagnosed cancer among women in the United States and Europe (1, 2). Despite the relative improvement in patient survival rates, breast cancer remains the most commonly diagnosed cancer and the second leading cause of cancer deaths in women worldwide. One of major challenges for the effective treatment of breast cancer is its intertumoral and intratumoral heterogeneity (3). Breast cancer can be initially classified into three different types based on the presence or absence of estrogen receptors (ERs), progesterone receptors (PRs), and the human epidermal growth factor receptor 2 (Her2/neu) (4). Hormone receptor-positive breast cancers that express ER and/or PR constitute approximately 60% of all breast cancers (5). The Her2/neu receptor is overexpressed in approximately 20% of all breast cancer cases; while TNBC constitute approximately 20% of breast cancer cases and are negative for the expression of ER, PR, and Her2/neu (6, 7).

Based on their molecular profile, breast cancers may also be clustered into basal-like and luminal subsets. Luminal breast cancers are more heterogeneous compared to basal cancers in terms of gene expression, mutation spectrum, copy number changes, and patient outcomes and can be further subdivided into luminal A and B subtypes (8, 9). The luminal A subtype represents 50–60% of breast cancer cases and is characterized by low histological grade and good prognosis. Luminal A cancers express ER and PR and have a low frequency of P53 mutations (9). Luminal B represents 10–20% of all breast cancers; compared with the luminal A subtype, these cancers are more aggressive; they have a higher grade, worse prognosis, and worse proliferative index. Luminal B display an increased expression of proliferation genes; they are ER+, PR+/−, Her-2+/−, and EGFR+ and have a higher frequency of P53 mutation (9). Because luminal cancers have a high frequency of PIK_3_CA mutations, the gene that encodes the p110α catalytic subunit of the phosphatidylinositol 3-kinase (PI_3_K), agents targeting the PI_3_K/AKT/mammalian target of rapamycin pathway may be useful for their treatment (10).

The basal-like subtype represents 10–20% of breast cancer cases. They are characterized by high proliferation, high histological grade, and poor prognosis. Basal-like cancers can be triple negative and have a high frequency of P53 mutations combined with loss of Rb1 (9, 11). However, not all basal-like cancers are triple negative; studies have shown that 5–45% of basal-like cancers express ER while 14% express Her2/Neu (12, 13). TNBC is a diverse group of malignancies and can be further categorized to different subtypes. An analysis of 21 breast cancer data sets containing 587 TNBC cases identified seven subtypes based on differential expression of a set of 2,188 genes: two basal like (BL1 and BL2), a mesenchymal (M), a mesenchymal-stem cell-like, an immunomodulatory, a luminal androgen receptor/luminal-like, and an unclassified type (14).

The deregulation of adult mammary stem cells (aMaSC) during tumorigenesis is believed to contribute to the development of TNBC. aMaSCs give rise to common progenitor cells that can differentiate either to basal progenitors that develop mature basal cells, or luminal progenitors. Disruption in the homeostasis of luminal progenitor cells may lead to the development of TNBC. Contributors in the development of TNBC include aberrantly activated signaling pathways, such as Wnt/β-catenin and Notch, transcriptional factors, like Snail, and embryonic stem cell markers including Sox2, Nanog, and Oct4. These alterations allow the restoration of proliferation capacity as well as the de-differentiation of these progenitor cells, leading to the accumulation of mutations that give rise to TNBC (15).

Traditionally, due to the lack of ER, PR, and Her2/Neu expression, the ineffectiveness of current breast cancer targeted therapies as well as due to the challenges in identifying key molecular drivers of TNBC progression, chemotherapy has been the foundation of treatment for patients with this disease over the last decades. Despite its sensitivity to chemotherapy, TNBC is associated with a higher risk of distant recurrence, high rates of metastases, higher probability of relapse and worse overall survival (OS) compared to other subtypes (16, 17).

## Complexity of TNBC Metastasis

**Figure 1:**
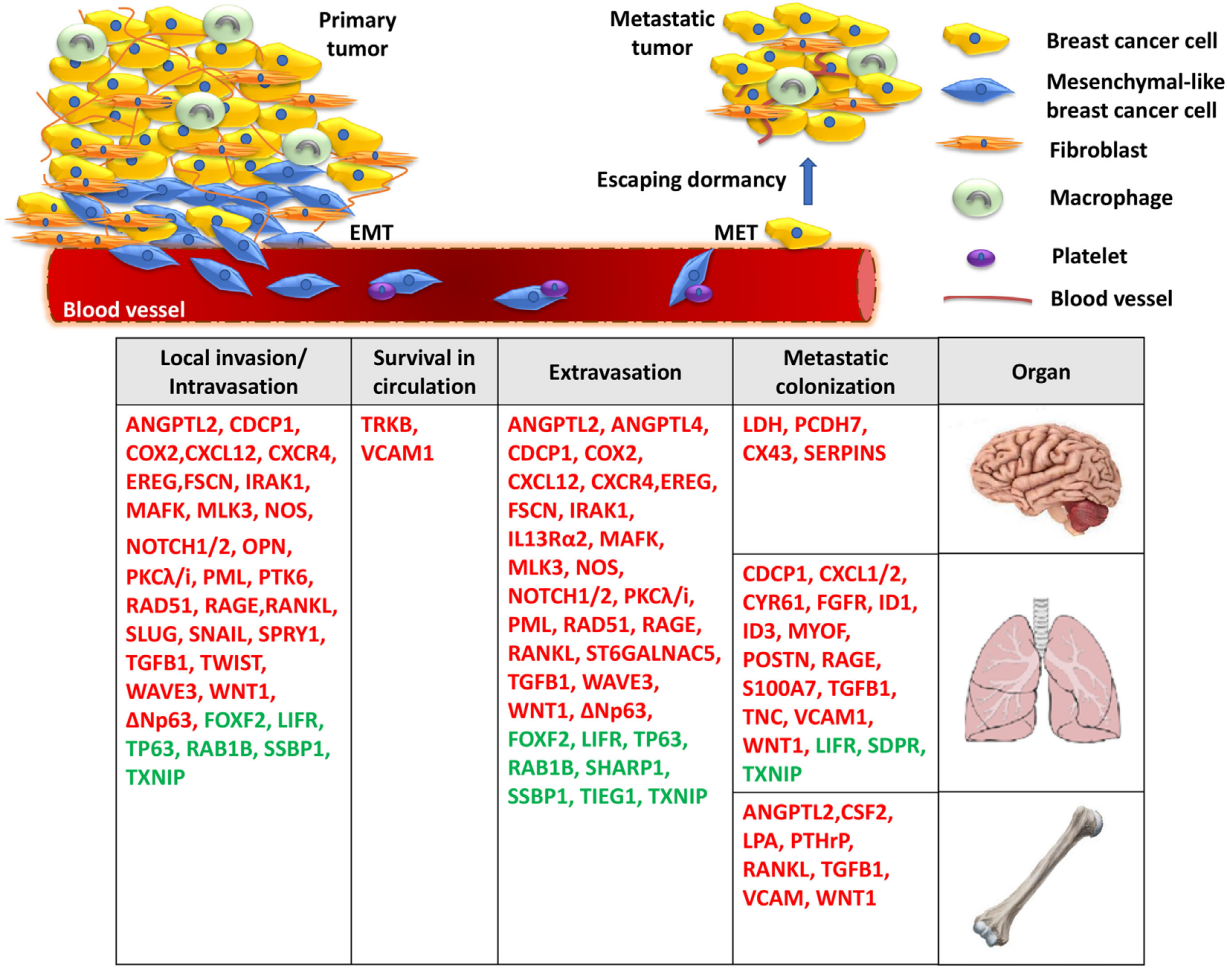
A model for the molecular basis of triple-negative breast cancer. During local invasion and intravasation, an epithelial-to-mesenchymal transition (EMT) transcriptional program is initiated along with the activation of matrix metalloproteases and pro-migratory signaling. Upon entering the circulation, breast cancer cells can interact with platelets, enable pro-survival pathways to suppress anoikis, and resist apoptotic signals. Then, migrated cancer cells extravasate through the endothelial blood vessel wall to a secondary organ where they enter a prolonged dormant state by forming micrometastases. Finally, the activation of metastasis-colonizing genes and the interaction with the local microenvironment create permissive conditions for macrometastatic outgrowth. Red: metastasis promoters, green: metastasis suppressors.

The dissemination of breast cancer cells and eventual metastatic growth to distant organs—predominantly the bone, lungs, and brain—represents a significant clinical problem, as metastatic disease is incurable and is the primary cause of death for the vast majority of TNBC patients. Metastatic spread of tumor cells is a highly complex, yet poorly understood process, and consists of multiple steps, including acquisition of invasive properties through genetic and epigenetic alterations, angiogenesis, tumor–stroma interactions, intravasation through the basement membrane, survival in the circulation, and extravasation of some cancer cells to distal tissues (18). However, disseminated cells that survive pro-apoptotic signals in their new environment often remain quiescent in secondary organs undergoing long periods of latency, also known as the dormancy period (19). It is well established that the outgrowth of metastatic cells in a foreign tissue microenvironment is a highly inefficient process and is considered as the rate-limiting step of breast cancer metastasis (20) (Figure 1). During this stage, breast cancer cells are usually difficult to detect and exhibit resistance to chemotherapy due to lack of proliferation (19). This remains a major clinical problem since patients, often considered as “survivors,” can develop metastatic disease years later. Disseminated tumor cells (DTCs) can enter a state of dormancy in secondary organs by exiting the proliferative cycle for an indefinite period or by achieving a balanced state of proliferation and apoptosis. Successful emergence from dormancy is the result of further evolution of surviving DTCs, by accumulating molecular alterations as well as *via* permissive interactions with the tumor microenvironment (19). By acquiring these characteristics, metastatic populations can optimally adapt to the host microenvironment and initiate colonization. While significant progress has been made to highlight some of the specific processes required for the breast tumor initiation, efforts have recently been focused on elucidating the roles of critical genes, the underlying molecular mechanisms and signaling pathways involved in the fatal late stages of metastatic dissemination. These studies are of outmost importance for the development of novel effective treatments against metastasis of TNBC.

## Genes Implicated in Multistep TNBC Metastasis

### Local Invasion/Intravasation

Upon accumulation of genetic and/or epigenetic alterations, breast cancer cells at the primary tumor initially acquire properties, such as self-renewal, ability to migrate, and invade the surrounding normal tissues. During local invasion, breast cancer cells undergo epithelial-to-mesenchymal transition (EMT), a highly orchestrated transcriptional program, initially described during embryonic development, associated with dramatic remodeling of cytoskeleton, loss of apico-basolateral polarity, dissolution of cell–cell junctions, concomitant with downregulation of epithelial markers and upregulation of mesenchymal genes (21). This process is triggered by EMT-master regulators, such as the transcription factors Slug, Snail, and Twist to promote TNBC cell migration and intravasation in the circulation (22–24). The TGFβ pathway plays a critical role in regulating this early metastatic event. During intravasation, TGFβ promotes overexpression of musculoaponeurotic fibrosarcoma oncogene family protein K (MAFK) to induce EMT and enhance tumor formation and invasion *in vivo* (25). The TGFβ-Smad signaling axis controls the EMT step in the malignant progression of breast cancer cells either by inducing the expression of master transcriptional regulators of EMT, as described above, or by epigenetic silencing of epithelial genes, including CDH1 (26). The EMT program regulated by TGFβ/Smad signaling also involves WAVE3, a WASP/WAVE family actin-binding protein. In TNBC cells, depletion of WAVE3 expression prevented TGFβ-induced EMT phenotype (27). However, despite numerous studies using cell lines and animal models suggesting a functional role of EMT and EMT-inducing transcription factors in promoting breast cancer metastasis, the *in vivo* role and clinical relevance of this process remains controversial (28–31).

Moreover, the majority of genes implicated in TNBC metastasis have been reported to play a major role at the initial stages of cancer cell dissemination which include migration, invasion, and intravasation. This is not surprising given the fact that cancer cell dissemination is thought to be an early event during breast cancer evolution and that primary and metastatic tumor growth is likely to progress in parallel (32). For example, activation of CXCR4 receptor *via* its ligand CXCL12 or ANGPTL2 was found to induce MLK3 and Erk1/2 signaling and promote intravasation which leads to the development of lung and bone metastases (33–39). This hyperactive signaling axis may also function in multiple stages of the metastatic cascade, including angiogenesis, extravasation, and osteolysis at the secondary organ. At the same time, it is becoming increasingly clear that trans-endothelial migration and invasion of breast cancer cells in the vasculature is inhibited by metastasis suppressors, including TP63, LIFR, lysyl oxidase-like 4 (LOXL4), FOXF2, SSBP1, RAB1B, and TIEG1 (25, 40–47), suggesting that the migratory and invasive potential of breast cancer cells is ultimately determined by the balance in the activity of these molecules. The identification of numerous genes implicated in the initial stages of TNBC metastasis highlights the significant challenges for early molecular diagnosis and therapy.

### Survival in Circulation

Upon entering the blood vessels, circulating tumor cells express proteins that have antiapoptotic and pro-survival functions which allow them to attach to and infiltrate specific secondary sites. Neurotrophic tyrosine kinase receptor TRKB was shown to inhibit anoikis, a form of cell death caused by lack of adhesion, *via* the PI_3_K/Akt pathway. These studies indicated that TRKB induces survival and proliferation of breast cancer cells to promote infiltration in the lymphatic and blood vessels and colonization in distant organs (48). In TNBC cells, brain-derived neurotrophic factor (BDNF) binds and activates TRKB receptor to regulate a network consisting of metalloproteases and calmodulin and thus modulate cancer–endothelial cells interaction. Importantly, Erk1/2 inhibitors were able to block the BDNF-induced phenotype, suggesting that blocking this pathway may be explored for therapeutic purposes against TNBC metastasis (49). In addition, the binding of platelets with circulating breast cancer cells has been shown to essential for their survival, evasion of pro-apoptotic signals, whereas interfering with this interaction inhibits the development of lung metastasis in TNBC mouse models (50, 51).

### Extravasation in Distal Sites

Many of the genetic alterations found to be involved in intravasation are also implicated in extravasation (Table 1) since, in large part, these two processes are considered “mirrored” to each other. The TGFβ pathway plays an important role in regulating both these metastatic steps. More specifically, TGFβ induces the assembly of a mutant-p53/Smad protein complex to inhibit the function of the metastasis suppressor TP63 and promote cell migration and invasion (40). During extravasation, TGFβ induces angiopoietin-like 4 (ANGPTL4) expression *via* the Smad signaling pathway; the increased levels of ANGPTL4 enhance the retention of cancer cells in the lungs by disrupting vascular endothelial cell–cell junctions, thus increasing the permeability of lung capillaries to facilitate trans-endothelial passage of breast cancer cells (52). Moreover, targeting the decoy interleukin-13 receptor alpha 2 (IL13Ra2) upregulates the metastasis suppressor TP63 in an IL13-mediated, STAT6-dependent manner and impairs extravasation of basal-like breast cancer cells to the lungs (41). Several reports also highlight the importance of the synergistic effects of genes in promoting metastasis by regulating specific stages of the process. For example, EREG, COX2, MMP1, and MMP2 can collectively promote metastatic extravasation to the lungs. These four genes were found to be overexpressed in TNBC cells independently of VEGF. Individual reduction of each gene or their silencing in different combinations produced limited effects on tumor growth *in vivo* while concurrent silencing of all four achieved nearly complete growth abrogation (53).

**Table 1:** List of genes involved triple-negative breast cancer metastasis.

Metastasis-promoting genes						
Gene	Function	Signaling pathway	Gene ontology	Stage	Organ site	Reference
ANGPTL2	Promotes osteolysis Migration Angiogenesis	Activates CXCR4 and Erk1/2 signaling	Receptor binding, extracellular space	Intravasation, extravasation Angiogenesis Micro- to macrometastasis colonization	Bone	(37)
ANGPTL4	Promotes trans-endothelial cancer cell migration by disrupting lung capillary cell junctions	Activated by TGFβ signaling	Angiogenesis	Extravasation	Lungs	(52)
CDCP1	Reduces lipid droplets, stimulates fatty acid oxidization and oxidative phosphorylation	Interacts with and inhibits acyl-CoA-synthetase ligase	Plasma membrane, protein binding	Intravasation, extravasation Metastatic colonization and growth	Lungs	(54)
COX2	Migration, invasion Promotes cancer stem cell maintenance	Mediates TGFβ-induced cancer cell stemness	Prostaglandin biosynthetic process, angiogenesis	Intravasation, extravasation Self-renewal	Bone	(53, 55–57)
CSF2	Osteoclast activation	Activated by NFκB signaling	Granulocyte macrophage colony-stimulating factor receptor binding	Micro- to macrometastasis colonization	Bone	(58)
CXCL1/2	Recruitment of myeloid cells	Activated by tumor necrosis factor-α/NFκB pathway	Receptor binding, extracellular region	Cancer cell survival at primary and metastatic sites	Lungs	(59, 60)
CXCL12	Binds CXCR4 to initiate downstream signaling	Activates CXCR4 signaling	Response to hypoxia, migration, endothelial cell proliferation, receptor binding	Intravasation, extravasation Angiogenesis	Lungs	(34)
CXCR4	Mediates actin polymerization and formation of lamellopodia Migration, Invasion Angiogenesis	Activated by ANGPTL2	Activation of MAPK activity, response to hypoxia, chemotaxis, G-protein coupled receptor activity	Intravasation, extravasation Angiogenesis	Lungs	(33–36)
CYR61	Vascularization	Activated by Sonic-Hedgehog/Gli1 signaling	Regulation of cell growth, angiogenesis	Angiogenesis Micro- to macrometastasis colonization	Lungs	(61)
EREG	Promotes vessel remodeling and invasion	VEGF-independent	MAPK cascade, angiogenesis	Intravasation Extravasation Angiogenesis	Lungs	(53)
FGFR	Suppresses apoptosis and promotes survival	Activates PI_3_K/Akt signaling	MAPK cascade, angiogenesis	Survival Primary tumor growth Micro- to macrometastasis colonization	Lungs	(62)
FSCN	Migration, invasion	Activates NFκB signaling Increases MMP2, MMP9 expression	Stress fiber, podosome, actin binding	Intravasation, extravasation	Lungs	(63, 64)
ID1, ID3	Promotes tumor re-initiation	Induced by NFκB-mediated IGF2/PI_3_K signaling	DNA binding transcription factor activity, angiogenesis	Micro- to macrometastasis colonization	Lungs	(65–67)
IL13Ra2	Migration	Suppresses IL13–STAT6–P63 signaling	Cytokine receptor activity, signal transducer activity	Extravasation	Lungs	(41, 60)
IRAK1	Invasion Promotes cancer stem cell maintenance	Activates NFκB and p38 signaling	Activation of MAPK activity, regulation of cytokine-mediated signaling	Intravasation, extravasation Self-renewal	Lungs	(68)
LDH	Catalyzes final reactions of glycolysis	Activates glycolytic pathway	Response to hypoxia, lactate dehydrogenase activity, lactate/pyruvate metabolism	Metastatic growth and colonization	Brain	(69, 70)
LPA	Produced by platelets to promote osteolysis	Induces interleukin-6 and IL8 secretion by breast cancer cells	Fibronectin binding, endopeptidase activity	Micro- to macrometastasis colonization	Bone	(71)
MAFK	Promotes epithelial-to-mesenchymal transition (EMT)	Activated by TGFβ pathway	DNA binding transcription factor activity	Intravasation, extravasation	Lungs	(72)
MLK3	Drives invasion and trans-endothelial migration	Mediates CXCL12/CXCR4 signaling to promote paxillin phosphorylation Increases FRA1, MMP1 and MMP9 levels	Activation of MAPK activity, protein serine/threonine kinase activity	Intravasation Extravasation	Lungs	(38, 39)
MYOF	Regulates lipid metabolism and mitochondrial function and promotes vesicle trafficking	Loss of MYOF suppresses AMPK phosphorylation and HIF1α stabilization due to metabolic stress	Phospholipid binding, plasma membrane, caveola	Metastatic growth and colonization	Lungs	(73)
NOS	Promotes EMT, self-renewal, migration, invasion	Activates TGFβ and hypoxia signaling	Response to hypoxia, nitric-oxide synthase activity	Intravasation, extravasation Self-renewal	Lungs	(74)
NOTCH1/NOTCH2	Migration, invasion Promotes cancer stem cell maintenance	Activate Notch signaling	Golgi membrane, cell fate determination, receptor activity	Intravasation, extravasation Tumor initiation and self-renewal	Lungs Bone	(75)
OPN	Mediates MSC-to-cancer-associated fibroblast transformation, tumor growth and invasion	Mediate TGFβ1 signaling to increase MMP2 and uPA levels	Osteoblast differentiation, cytokine activity	Tumor growth Invasion	Lung Liver	(76, 77)
PCDH7/CX43	Promotes cancer cell-astrocyte interaction	Activates IFNγ, NFκB pathway	Calcium ion binding, plasma membrane, cell adhesion	Micro- to macrometastasis colonization	Brain	(78)
PKCλ/i	Migration, invasion	Activated by TGFβ/IL1β Activates NFκB	Golgi membrane, protein serine/threonine kinase activity	Intravasation, extravasation	Lungs	(79)
PML	Migration, invasion	Activated by hypoxia/HIF1α signaling	Response to hypoxia	Intravasation, extravasation	Lungs	(80)
POSTN	Expressed by stromal or cancer cells Promotes cancer stem cell maintenance	Activates Wnt1 and Wnt3A signaling Activates NFκB and Erk signaling	Negative regulation of cell–matrix adhesion, response to hypoxia	Micro- to macrometastasis colonization	Lungs	(81, 82)
PTHLH	Osteoclast activation	Activated by TGFβ signaling Induced by miR-218-5p	Osteoblast development, hormone activity	Micro- to macrometastasis colonization	Bone	(83, 84)
PTK6	Promotes EMT *via* Snail upregulation	Activates EGF and PI_3_K/Akt signaling	Protein tyrosine kinase activity	Local invasion Intravasation	Lungs	(85, 86)
RAD51	Promotes aberrant DNA repair	Double-strand break repair pathway	Double-strand break repair *via* homologous recombination	Intravasation, extravasation	Lungs	(87)
RAGE	Binds S100A7 to promote recruitment of tumor-associated macrophages and migration	Activates Erk and NFκB pathways	Cytokine production, inflammatory responses	Primary and metastatic tumor growth Intravasation, extravasation	Lungs	(88)
RANKL	Migration Osteoclast activation	Activates NFκB signaling Induced by miR-218-5p	Osteoblast proliferation, cytokine activity, monocyte chemotaxis	Intravasation, extravasation Micro- to macrometastasis colonization	Bone	(84, 89)
S100A7	Promotes inflammation, recruitment of tumor-associated macrophages and angiogenesis	Activates STAT3, Akt and Erk pathways	Response to ROS, angiogenesis	Primary and metastatic tumor growth	Lungs	(90)
SERPINS (NS, B2, D1)	Inhibit plasminogen activation Promote vascular co-option	Inhibits FasL-mediated apoptotic pathway	Serine-type endopeptidase inhibitor activity, chemotaxis, blood coagulation	Survival Micro- to macrometastasis colonization	Brain	(91)
SLUG	Promotes EMT Migration Invasion Survival by suppressing Puma-induced apoptosis	Activated by Erk, FGF signaling Activates TGFβ signaling	EMT	Local invasion Intravasation Metastatic colonization	Lungs	(22, 92–94)
SNAIL	Promotes EMT Migration Invasion	Activated by EGF signaling Activates TGFβ signaling	EMT, Mesoderm formation	Local invasion Intravasation	Lungs	(23, 94–96)
SPRY1	Promotes EGFR stability Promotes EMT, migration, invasion	Activates EGFR signaling	Mitotic spindle orientation	Intravasation, extravasation	Lungs	(97)
ST6GALNAC5	Mediates brain infiltration across the blood–brain barrier	Catalyzes cell-surface sialylation	Golgi membrane, sialytransferase activity	Extravasation	Brain	(98)
TGFβ1	EMT Migration Invasion Promotes osteoclastic bone resorption	Activates AP1- and Smad4-dependent interleukin-11 and CTGF expression. Maintains Smad2-dependent, DNMT1 mediated DNA methylation and silencing of CDH1	EMT, vasculogenesis, neural tube closure, response to hypoxia	Intravasation, extravasation Colonization	Lungs Bone	(26, 99, 100)
TNC	Promotes survival and outgrowth of macrometastases	Activates Notch and Wnt signaling	Osteoblast differentiation, extracellular region	Micro- to macrometastasis colonization	Lungs	(101)
TRKB	Suppresses anoikis to promote survival in circulation Modulates breast cancer-endothelial cell interaction	Interacts with brain-derived neurotrophic factor ligand Activates Erk and PI_3_K signaling	Vasculogenesis, neuron migration	Survival in circulation	Lungs Bone	(48, 49)
TWIST	Promotes EMT Migration Invasion	Induced by Wnt signaling	Neuron migration, neural tube closure, morphogenesis	Local invasion Intravasation	Lungs	(24, 102)
VCAM1	Osteoclast activation through interaction with integrin α4β1 Binds metastasis-associated macrophages via α4 integrins	Activated by NFκB pathway Activates PI_3_K/Akt pathway	Inflammatory response, integrin binding, extracellular space	Survival Micro- to macrometastasis colonization	Bone Lungs	(60, 103, 104)
WAVE3	Promotes EMT	Activates TGFβ signaling	Actin binding, cytoskeleton organization, lamellipodium	Intravasation, extravasation	Lungs	(27)
Wnt1	Maintains CSC renewal Migration Invasion	Activates Wnt/β-catenin signaling Induced by miR-218-5p	Embryonic axis specification, frizzled binding, cytokine activity	Intravasation, extravasation Colonization	Lungs Bone	(84, 105–107)
ΔNp63	Promotes migration, invasion EMT	Activates PI3K signaling and CD44v6 expression	Transcription factor activity, p53 binding	Intravasation, extravasation	Lungs Bone	(108)
**Metastasis suppressor genes**						
FOXF2	Inhibits migration, invasion	Blocks EMT by suppressing Twist	Transcription factor activity, EMT	Intravasation, extravasation	Lungs	(44)
LIFR	Inhibits migration, invasion	Targeted by miR-9 Activates Hippo/YAP pathway	Regulation of cytokine-mediated signaling pathway	Intravasation, extravasation Metastatic colonization	Lungs	(43)
LOXL4	Inhibits migration, invasion, primary and metastatic tumor growth	Suppresses collagen synthesis	Scavenger receptor activity, oxidoreductase activity	Intravasation, extravasation	Lungs	(25)
TP63	Inhibits migration, invasion Regulates miRNA processing	Inhibited by TGFβ-induced Smad/mutant-p53 complex Induced by IL13 Upregulates Dicer to control miRNA processing	Transcription factor activity, p53 binding	Intravasation, extravasation	Lungs	(40–42)
RAB1B	Inhibits migration, invasion	Activates TGFβ/Smad signaling	Golgi membrane	Intravasation, extravasation	Lungs	(46)
SDPR	Inhibits extravasation, Apoptosis	Silenced by DNA methylation Suppresses NFκB, Erk	Phosphatidylserine binding	Extravasation Apoptosis at secondary organ	Lungs	(109)
SHARP1	Promotes degradation of hypoxia-inducible factors Inhibits migration, invasion	Suppresses hypoxia-inducible pathway	DNA binding transcription factor activity	Extravasation	Lungs	(110)
SSBP1	Inhibits TGFβ-induced EMT	Regulates mitochondrial retrograde signaling	Single-stranded DNA binding, RNA binding, mitochondrial matrix	Intravasation, extravasation	Lungs	(45)
TIEG1	Inhibits migration, invasion	Downregulates EGFR expression to suppress EGF signaling	DNA binding transcription factor activity	Intravasation, extravasation	Lungs	(47)
TXNIP	Blocks glucose uptake and aerobic glycolysis Suppresses EMT	Suppressed by Myc oncogene and miR-373	Mitochondrial intermembrane space, enzyme inhibitor activity	Intravasation, extravasation Metastatic colonization and growth	Lungs	(111, 112)

*A comprehensive list of genes implicated in various stages of the metastatic cascade, their reported functions, upstream or downstream regulatory signaling pathways involved, gene ontology, as well as the secondary organs which become affected*.

### Metastatic Colonization

Following extravasation and infiltration at the secondary site, a genetic program is initiated so that cancer cells can escape dormancy and form micro and macrometastatic tumors. Initially, EMT plasticity and the reversal to MET phenotype have been shown to be important for metastatic colonization (113). During this process, epithelial phenotype becomes re-established through miR-200-mediated downregulation of ZEB1, SIP1 to promote metastatic colonization (114, 115). Also, breast DTCs in the bone marrow gain the ability to form typical osteolytic metastases by producing parathyroid hormone-related protein (PTHLH), tumor necrosis factor-α (TNFα), interleukin-6 and/or interleukin-11. These factors stimulate the release of receptor activator of nuclear factor-κB ligand (RANKL) from osteoblasts which induces osteoclast formation (33, 58, 83, 116). Furthermore, inflammation in the lung microenvironment could also be responsible for triggering the escape of metastatic breast cancer cells from latency leading to metastatic colonization (117). A subset of genes contributing to primary tumor growth can also promote survival and growth at the secondary site. Chemokines CXCL1/2 mediate chemoresistance and lung metastasis by attracting myeloid cells into the tumor, which produce low molecular weight calcium-binding proteins S100A8/9 that enhance cancer cell survival by binding to the receptor for advanced glycation end products (RAGE) (59). Another calcium binding protein, S100A7 has been found to enhance tumor growth and metastasis, by binding to RAGE and activating Erk and NFκB signaling (88, 90). Furthermore, fibroblast growth factor receptor (FGFR) was shown to trigger pro-survival signals through PI_3_K/Akt signaling and promote outgrowth of metastatic breast cancer cells to the lungs (62). However, it needs to be highlighted that cellular and genetic context among cancers influences whether proteins act as tumor suppressors or metastasis promoters. One controversial example is LOXL4 which has been shown to recruit bone marrow-derived cells and facilitate colonization of TNBC to the lungs *via* a HIF1α-dependent mechanism (118). However, in another study, knockdown of LOXL4 expression in TNBC cells promoted primary tumor growth and lung metastasis which was associated with thickening of collagen bundles and remodeling of the extracellular matrix (ECM) within tumors (25). Overall, it is noteworthy that while some genes have been associated only with TNBC metastasis so far (i.e., TIEG1, MAFK, MLK3, SDPR), the majority is also involved in other tumor types, suggesting a more fundamental role in cancer progression.

## Concluding Remarks on Current and Future Perspectives on TNBC Metastasis Therapy

Due to their molecular heterogeneity, there are no drugs that can target the entire spectrum of TNBC tumors and each subtype is vulnerable to specific therapeutic approaches. Despite the lack of FDA-approved targeted therapies for TNBC to date, ongoing clinical trials are assessing the efficacy of single or combinatorial approaches that tackle different TNBC molecular alterations. Up to 20% of TNBC have been associated with germ-line mutations in BRCA1 (119). TNBC tumors with loss of function of BRCA1 or BRCA2 are sensitive to poly(ADP-ribose) polymerase inhibitors and alkylating agents that induce DNA double-strand breaks (120). Olaparib has been the most successful PARP inhibitor against BRCA-mutated TNBC, inducing partial responses in 54% of patients when administered as a single agent (121) and an overall response rate of 88% when combined with carboplatin (122). Anti-androgens as well as FGFR inhibitors have been tested in clinical trials against TNBCs that are androgen receptor-positive or harbor FGFR amplification, respectively (123, 124). Gamma-secretase inhibitors that block the NOTCH pathway are currently in clinical trials for TNBC patients with upregulated NOTCH signaling (125). All together clinical trials have shown that each agent alone provides small or no benefit in TNBC patients suggesting that further effort is needed to discover novel targets of TNBC and to identify each patient’s molecular profile that will lead to a more individualized treatment.

Toward this goal, some of the metastasis-promoting genes reported here could be further exploited for the future development of promising targeted therapies. Since local invasion, intravasation and possibly extravasation are thought to occur relatively early in the metastatic process (32), a plausible strategy would be to target dormancy and the outgrowth of macrometastatic tumors in distal organs. Since this final stage is considered the critical “rate-limiting” step of the “invasion-metastasis” cascade requiring even years to be completed, it provides a window of opportunity for effective therapy. Therefore, different approaches could aim against “druggable” molecules that facilitate metastatic colonization, such as overexpressed receptors or secreted molecules (i.e., CXCL1/2, FGFR, TGFβ1, WNT1, ANGPTL2, CSF2, RANKL), which target commonly deregulated signaling networks at this late-stage (Table 1). Ongoing clinical trials are evaluating the efficacy of the TGFβR1 inhibitor LY2157299 with paclitaxel (NCT02672475), whereas the FGFR inhibitor Lucitanib is also under testing (NCT02202746) for patients with metastatic TNBC. The ultimate goal would be, if not to completely eliminate dormant metastatic breast cancer cells, to prolong dormancy period and hopefully transform this stage into a chronic inactive cancer cell state.

Importantly, recent studies have shown that tumor cells are able to evade immune responses by activating negative regulatory pathways, also known as immune checkpoints, that block T-cell activation through cytotoxic T-lymphocyte protein 4 (CTLA4) or *via* binding of the programmed cell death protein 1 (PD1) receptor expressed on T-cell surface to the PDL1 ligand expressed by cancer cells in response to various cytokines (126). The recent development and FDA approval of anti-CTLA4, anti-PDL1, and anti-PDL1 monoclonal antibodies that elicit antitumor clinical responses in a variety of solid cancers created enthusiasm for cancer therapy (127). Currently, several clinical trials are underway to evaluate the efficacy of this approach in TNBC as well (128).

However, a major clinical problem is that breast cancer is considered one of the most desmoplastic tumor types due to the production of excessive amounts of ECM components, such as collagen and hyaluronan, which generate mechanical stresses within the growing tumor (129). This results in blood vessel compression, hypoperfusion, and hypoxia which promote cancer progression and metastasis as well as hinder drug delivery (130). Therefore, targeting components of the tumor microenvironment has also been recently proposed as another promising strategy for TNBC therapy by improving tumor penetration and delivery of cytotoxic drugs (131). For example, targeting of cancer-associated fibroblasts using pirfenidone, an FDA-approved drug for idiopathic pulmonary fibrosis, has been shown to suppress metastasis of TNBC in combination with doxorubicin (132). This effect is likely to be mediated through remodeling of tumor microenvironment which reduces ECM components through suppression of TGFβ signaling, improves perfusion and delivery of chemotherapy (133). Similar effects have also been demonstrated using the anti-fibrotic drug Tranilast or the anti-hypertensive drug Losartan in combination with chemotherapy or nanotherapy in mouse models for TNBC (134–136).

In conclusion, this evidence suggests that efforts in the near future should be focused toward the development and testing of novel anti-metastatic targeted therapies for late-stage TNBC that could be used in combination with existing chemotherapies, immunotherapies as well as with microenvironment-remodeling agents that can improve drug penetration and overall therapeutic efficacy.

## Author Contributions

CN and PB wrote the paper and helped with illustrations. PP conceived the theme, wrote the paper, and prepared illustrations.

## Conflict of Interest Statement

The authors declare that the research was conducted in the absence of any commercial or financial relationships that could be construed as a potential conflict of interest.
